# Based on multiple machine learning to identify the ENO2 as diagnosis biomarkers of glaucoma

**DOI:** 10.1186/s12886-022-02350-w

**Published:** 2022-04-02

**Authors:** Min Dai, Zhulin Hu, Zefeng Kang, Zhikun Zheng

**Affiliations:** 1 From the Department of Ophthalmology, Yunnan University Affiliated Hospital, Yunnan, 650021 China; 2 From the Eye Hospital of China Academy of Traditional Chinese Medicine, Beijing, 100040 China

**Keywords:** Diagnostic markers, Logistic regression, Random forest, Lasso regression

## Abstract

**Purpose:**

Glaucoma is a generic term of a highly different disease group of optic neuropathies, which the leading cause of irreversible vision in the world. There are few biomarkers available for clinical prediction and diagnosis, and the diagnosis of patients is mostly delayed.

**Methods:**

Differential gene expression of transcriptome sequencing data (GSE9944 and GSE2378) for normal samples and glaucoma samples from the GEO database were analyzed. Furthermore, based on different algorithms (Logistic Regression (LR), Random Forest (RF), lasso regression (LASSO)) two diagnostic models are constructed and diagnostic markers are screened. GO and KEGG analyses revealed the possible mechanism of differential genes in the pathogenesis of glaucoma. ROC curve confirmed the effectiveness.

**Results:**

LR-RF model included 3 key genes (NAMPT, ADH1C, ENO2), and the LASSO model outputted 5 genes (IFI16, RFTN1, NAMPT, ADH1C, and ENO2), both algorithms have excellent diagnostic efficiency. ROC curve confirmed that the three biomarkers ADH1C, ENO2, and NAMPT were effective in the diagnosis of glaucoma. Next, the expression analysis of the three diagnostic biomarkers in glaucoma and control samples confirmed that NAMPT and ADH1C were up-regulated in glaucoma samples, and ENO2 was down-regulated. Correlation analysis showed that ENO2 was significantly negatively correlated with ADH1C (cor = -0.865714202) and NAMPT (cor = -0.730541227). Finally, three compounds for the treatment of glaucoma were obtained in the TCMs database: acetylsalicylic acid, 7-o-methylisomucitol and scutellarin which were applied to molecular docking with the diagnostic biomarker ENO2.

**Conclusions:**

In conclusion, our research shows that ENO2, NAMPT, and ADH1C can be used as diagnostic markers for glaucoma, and ENO2 can be used as a therapeutic target.

**Supplementary Information:**

The online version contains supplementary material available at 10.1186/s12886-022-02350-w.

## Introduction

Glaucoma is a group characterized by progressive degeneration of retinal ganglion cells of optic neuropathy, which is the main cause of blindness and raises the prevalence [1, 2]. There are four categories of adult glaucoma: primary open-angle (POAG) and closed-angle glaucoma, and secondary open-angle (OAG) and closed-angle glaucoma (ACG). Compared with normal, primary glaucoma has neuropathy and elevated intraocular pressure. Secondary glaucoma has some causes of elevated intraocular pressure, including trauma, neovascularization, pigment dispersion, inflammation or pseudodefoliation. The diagnosis and classification of glaucoma need to combine clinical examination, intraocular pressure measurement, visual field, and structural imaging parameters [2, 3].

Biomarkers make it more convenient to explore the ocular matrix. Repeated sampling and evaluation of biomarkers can show the biological process of disease progression and drug treatment [4]. Studies have reported that small molecules to macromolecules, nucleic acids to proteins can be used as molecular biomarkers. At present, biomarkers in the tear film, aqueous humor, vitreous, and blood/serum have been proved to be useful in the diagnosis of glaucoma [2].

Current used treatment includes drugs, laser surgery and minimally invasive surgery for glaucoma [5]. Glaucoma treatment technology were relatively mature, but early detection and treatment was vital for excellent vision prognosis of glaucoma. Patients have lost 35 ~ 40% of retinal ganglion cells when glaucoma was diagnosed clinically, which was a common phenomenon [6]. The glaucoma was asymptomatic in the immediate stage and onset of symptoms in a relatively late stage, so the diagnosis was often delayed, Which was causing irreversible damage to patients and was the biggest challenge to diagnosis of glaucoma [7].

The discovery and application of glaucoma biomarkers can be difficult, the marker selection is limited by patients with individual differences, techniques for the use, and analysis of therapeutic drugs [8]. At present, it is possible to provide a comprehensive ophthalmology examination by suspicious patients with positive family history or results of optic nerve papilla examination, which can provide convenience for glaucoma diagnosis [9]. Previous studies have reported that hundreds of candidate biological markers have been proposed (> 450). However, biomarker diagnosis has not yet entered the clinical stage due to the sensitivity and specificity of the difference in the patient's genetic, sick and therapeutic phase [2]. Therefore, the candidate glaucoma biomarker will use to transform into clinical practice that still takes a lot of research.

This study obtains glaucoma clinical data from the public database GEO database. a large number of data analysis were used which including the obtained of database sample genes and the prediction of targeted biomarkers and their transcription factors in the online database. In addition, through the molecular docking between the protein crystal encoded by the diagnosed biomarker and the small molecule drug, it is tested that the targeted drug can be successfully docked with the marker protein. It provides a reference for biomarkers to become the key target of targeted drug therapy in the future.

## Methods

### Data sources

The GEO database dataset GSE9944, GSE2378 were used to download the gene sequencing data and sample information of glaucoma and normal samples. GSE9944 selected data from the GPL8300 platform with a large number of samples, including 13 glaucoma samples and 6 normal samples. GSE2378 selected the data measured by the GPL8300 platform with a large number of samples, including 7 glaucoma samples and 6 normal samples.

### Analysis methods

#### Differentially expressed genes analysis and annotation

In our study, we used the limma package of R to compare the differences of gene expression levels between glaucoma / normal sample groups. The screening conditions are: < 0.05 and丨log2FC丨 > 0.5.The VennDiagram package was used to compare the two data sets up and down-regulated differential express genes, and the differential expression genes commonly discovered by the two data sets. Differential expression gene function was analyzed (Including GO function analysis and KEGG pathway enrichment analysis, *P* < 0.05 was significant) by the Cluster Profiler (version 3.18.0) package. GO plot was used to structure the chord diagram of GO items, and Ggplot2 package was used to create the volcano diagram (differential genes) and bubble diagram (KEGG pathway) [10–12]. Differential expression analysis aims to find out the genes with significant differences in expression between glaucoma and normal sample groups. The grouping may be different biological states, such as drug treatment and control, diseased individuals and healthy individuals, different tissues, different development stages, etc.

#### Construction of glaucoma diagnostic model and screening of diagnostic markers

Machine algorithms Logistic Regression (LR), Random Forest (RF), and lasso regression (LASSO) were used to construct the diagnostic model of glaucoma based on the differentially expressed genes of glaucoma. Based on LR-RF and LASSO algorithm, the RandomForest and Glmnet packages of the language of R were used to construct glaucoma diagnosis models, and the AUC values of each model were calculated respectively. ROC curves and AUC values were used to evaluate the effectiveness and accuracy of the model. The RMS package of diagnostic biomarkers was used to build a nomogram and straightening curve to assess the clinical risk of glaucoma patients.

#### Analysis of transcription factors regulatory network and noncoding RNA regulatory network

The network analyst database was used to predict the transcription factors of biomarkers and obtain the gene—transcription factor (TF) pairs. The mirwalk database was used to predict miRNA targeting diagnostic biomarkers. The Starbase was applied to predict the targeting relationship of miRNA-lncRNA (Supplementary materials 1). The relationship between the biomarker’s gene-TF network and the predicted miRNA—lncRNA targeting was visualized by Cytoscape (version 3.8.0).

#### The expression and correlation analysis of glaucoma diagnostic biomarkers

Ggplot2 (version 3.3.2) and Ggpubr (version 0.4.0) packages of R were used to create scatter plots, which show the expression of diagnostic biomarkers in normal and glaucoma samples. Rank-sum test was used to compare the expression of glaucoma biomarkers between the two groups. The Ggcorrplot software package was used to analyze the relationships between three diagnostic markers for glaucoma. ROC curves constructed based on the GSE2378 and GSE9944 datasets were used to evaluate and verify the diagnostic efficiency of three glaucoma diagnostic markers.

#### Molecular docking analysis of diagnostic markers and compounds with therapeutic activity for glaucoma

Gene cards database was used to predict the disease targets of glaucoma. The TCMSP database was used to obtain active compounds that have therapeutic effects on glaucoma. The PDB database was used to download protein structure data of glaucoma biomarkers. The small and water molecules included in the original protein structure were completely removed by Auto Dock Tools before molecular docking, as well as the calculation of hydrogen bonds and charges. The PubChem database was used to download the structure of compounds with glaucoma therapeutic activity, which was predicted by Auto Dock Tools for charge balance and rotatable covalence bonds. The center of activity was then determined according to the range of the receptor docking box. Finally, Auto Dock Vina was used to calculate the docking of receptors and ligands, the structure with the lowest binding free energy (the highest binding affinity) in the output results was selected, and PyMoL software was used to visualization and beautification.

## Results

### Screening and functional annotation analysis of differential expression genes in glaucoma

**Fig. 1:**
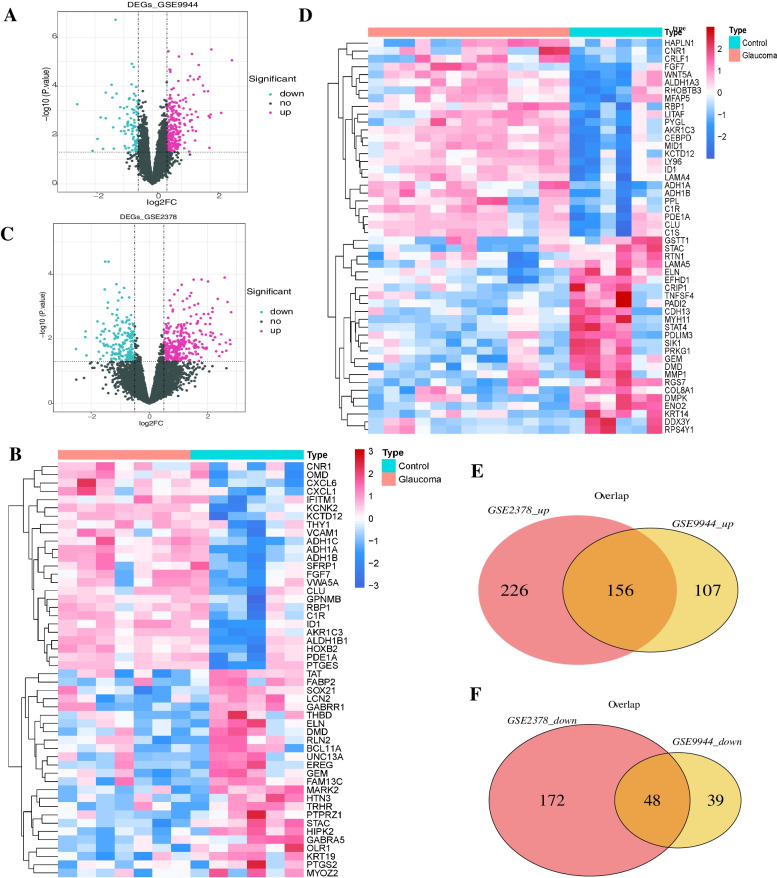
Screening and analysis of glaucoma-related genes. **A** Volcanic map of differentially expressed genes between GSE9944 glaucoma and normal samples (red: up, blue: down. The dotted line on the horizontal and vertical axis indicates that the absolute threshold of logFC is 0 and the *p*—value threshold is 0.05, respectively); **B** Heat map of differentially expressed genes between GSE9944 glaucoma and normal samples (Said every little squares one gene, its color amount, said the gene expression to express the greater the amount of the deeper the color (red: up, blue: down)); **C** Volcanic map of differentially expressed genes between GSE2378 glaucoma and normal samples; **D** Heat map of differentially expressed genes between GSE9944 glaucoma and normal samples; **E**: Screening of up-regulated glaucoma related genes (Red: GSE2378 increase expression of genes, yellow: GSE9944 increase expression of genes); **F**: Screening of down regulated glaucoma related genes (Red: GSE2378 down expression of genes, yellow: GSE9944 down expression of genes)

Differential gene analysis indicated that the 350 and 478 differentially expressed genes were obtained in GSE9944 and GSE2378 data sets (Supplemental Tables 1 and 2 for the statistical table of clinical information), respectively. Among them, 263 genes were up-regulated and 87 genes down-regulated in glaucomatous tissue samples of GSE9944 data sets (Fig. 1A), 301 genes were up-regulated and 177 genes down-regulated in glaucomatous tissue samples of GSE2378 data sets (Fig. 1C). The top50 of differentially expressed gene expression in GSE9944 and GSE2378(Fig. 1B and 1D). The intersection of differentially expressed genes between the two groups showed that 156 genes were up-regulated and 48 genes were down-regulated (Fig. 1E, F).

**Fig. 2:**
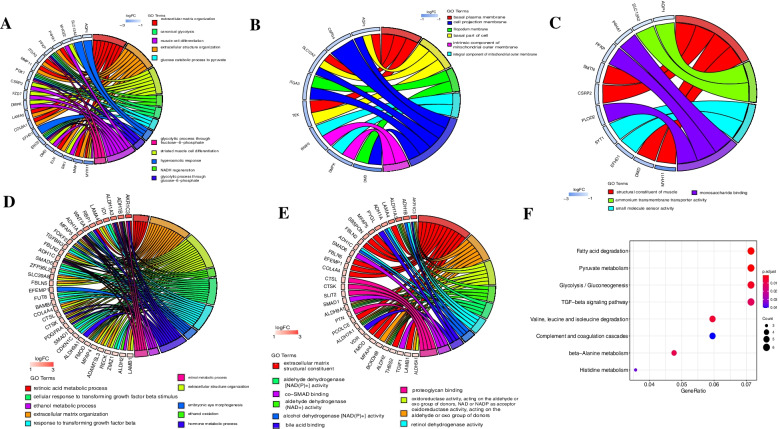
Functional enrichment of differentially expressed genes and enrichment results of KEGG pathway down-regulated differentially expressed genes, **A** biological process (BP); **B** cellular components, (CC), **C** molecular functions (MF). Increase the function of differentially expressed genes, **D** BP, **E** MF. The left is the up-regulation of differential expression genes, and the right side is different colors represent different pathways; the connection indicates the relationship between genes and pathways, and the width of the connection indicates the strength of the relationship. **F** Up-regulating differential expression gene’s KEGG path enrichment results (The bubble size indicates how much passage gene, the color of the color from blue to red is from low to high)

GO enrichment results show that the main enrichment cleavage the differentially expressed genes of glucose metabolism, muscle cell differentiation and NADH regeneration and other biological processes: such as extracellular matrix organization, extracellular structure organization, striated muscle cell differentiation, NADH regeneration, canonical glycolysis, glucose catabolic process to pyruvate, hyperosmotic response, glycolytic process through glucose-6-phosphate, muscle cell differentiation and glycolytic process through fructose-6-phosphate, etc., (Fig. 2A).

In addition, the down-regulated differentially expressed genes are mainly significantly related to muscle structural components, small molecule sensor activity, monosaccharide binding in terms of molecular function, including structural constituent of muscle, ammonium transmembrane transporter activity, small molecule sensor activity, monosaccharide binding (Fig. 2B). In terms of cell composition, it is significantly related to the cell basement membrane, cell base, filamentous membrane, and mitochondrial outer membrane. Which including basal plasma membrane, basal part of a cell, filopodium membrane, an integral component of the mitochondrial outer membrane, cell projection membrane and intrinsic component of the mitochondrial outer membrane (Fig. 2C).

The up-regulated differentially expressed genes are significantly related to hormone metabolism, retinoic acid metabolism, small molecule metabolism, retinol metabolism, and microglia activation in biological processes, including retinoic acid metabolic process, extracellular matrix organization, extracellular structure organization, ethanol oxidation, cellular response to transforming growth factor-beta stimulus, response to transforming growth factor-beta, embryonic eye morphogenesis, hormone metabolic process, ethanol metabolic process, retinol metabolic process, etc., (Fig. 2D). In terms of molecular function, it is significantly related to retinol dehydrogenase activity, oxidoreductase activity, and proteoglycan binding, including proteoglycan binding, acting on the aldehyde or oxo group of donors, oxidoreductase activity, acting on the aldehyde or oxo group of donors, NAD or NADP as acceptor, aldehyde dehydrogenase (NAD +) activity, aldehyde dehydrogenase [NAD(P) +] activity, retinol dehydrogenase activity, alcohol dehydrogenase [NAD(P) +] activity, bile acid-binding, co-SMAD binding, proteoglycan binding, etc., (Fig. 2E). In terms of cell composition, it is significantly related to the collagen-containing extracellular matrix.

KEGG (Kyoto Encyclopedia of Genes and Genomes, KEGG) pathway enrichment analysis found that there was no down-regulated differentially expressed gene related pathway, while the up-regulated differentially expressed genes were significantly related to amino acid metabolism, glucose metabolism, and fatty acid metabolism, including Histidine metabolism, beta-Alanine metabolism, Complement, and coagulation cascades, Valine, leucine and isoleucine degradation, TGF -beta signaling pathway, Glycolysis/Gluconeogenesis, Pyruvate metabolism, Fatty acid degradation (Fig. 2F).

### Diagnostic models and biomarkers of glaucoma created by Logistic Regression, Random Forest, and LASSO regression

**Fig. 3:**
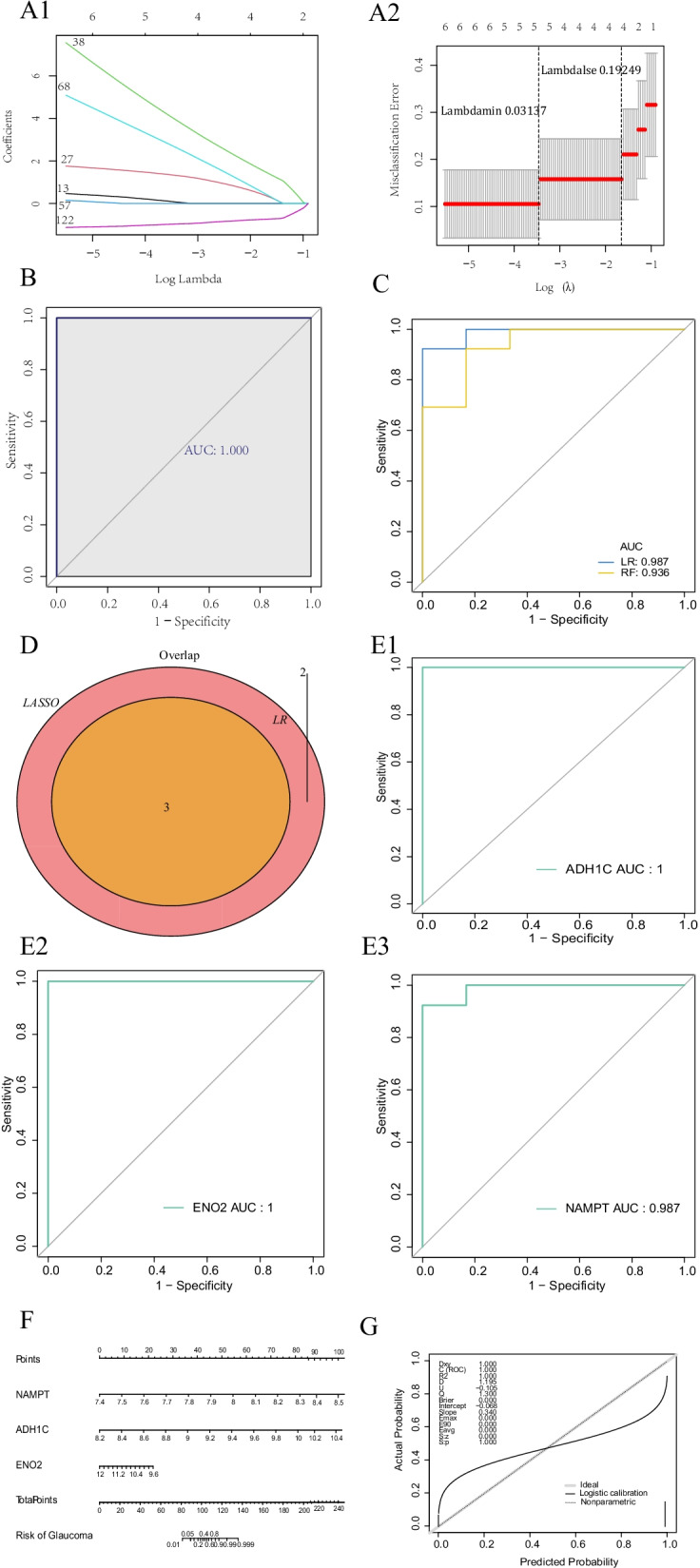
Three machine algorithms construct and analyses the best diagnostic model. **A** Lasso regression analysis Screening feature gene (A1: The horizontal coordinate deviance represents the ratio of the residual explanation, and the ordinate is the coefficient of the gene; A2: the abscissa is log (Lambda), The ordinate represents the error of cross-validation, in practice, the left dashed position is the smallest position of the cross-validation error, according to this location (lambda.) MIN) Determine the corresponding abscissa log (Lambda), the upper side shows the number of feature genes, find the optimal log (Lambda) value, find the corresponding gene and its coefficients in the left, and the residual explanation of the model proportion). **B** Evaluation of LASSO diagnostic model by ROC curve. **C** ROC curve of LR and RF diagnosis model of assessment. **D** Intersection of LASSO characteristic gene and LR_RF characteristic gene. **E** Diagnostic biomarkers (E1: ADH1C, E2: ENO2, E3: NAMP) of ROC curve. **F** Nomogram of diagnostic biomarkers (ADH1C, ENO2, NAMP). **G** Nomogram of calibration curve

In this part, the Glmnet and random Forest packages respectively applied LASSO and LR-RF algorithms to construct glaucoma diagnostic models. When λ min was 0.03137, the glaucoma diagnostic model derived from LASSO regression was optimal (Fig. 3A). The model contained five genes including IFI16, RFTN1, NAMPT, ADH1C, and ENO2, and the AUC value of the ROC curve was 1 (Fig. 3B), which proved the accuracy and sensitivity of the model. The AUC values of the LR-RF diagnostic model were 0.987 and 0.936 respectively (Fig. 3C). The genes output by the model were NAMPT, ADH1C, and ENO2. The gene intersection results of the LASSO regression model and LR-RF model indicated that three genes NAMPT, ADH1C, and ENO2 could be used as biomarkers in this study (Fig. 3D).

The diagnostic efficiency of the three gene evaluated by the ROC curve confirmed that the AUC values of the three genes were all 1, which indicates that ADH1C, ENO2, and NAMPT are biomarkers for the diagnosis of glaucoma (Fig. 3E). Furthermore, the nomogram was constructed for the three diagnostic biomarkers (Fig. 3F). The C value of the nomogram correction curve was 1, and the calibration curve indicated that the prediction effect of the Neglect diagram is excellent (Fig. 3G). These results suggest that the combined expression levels of the three genes can be used to diagnose glaucoma.

### Transcription factor regulatory network and lncRNA-miRNA-mRNA regulatory network of diagnostic biomarkers

**Fig. 4:**
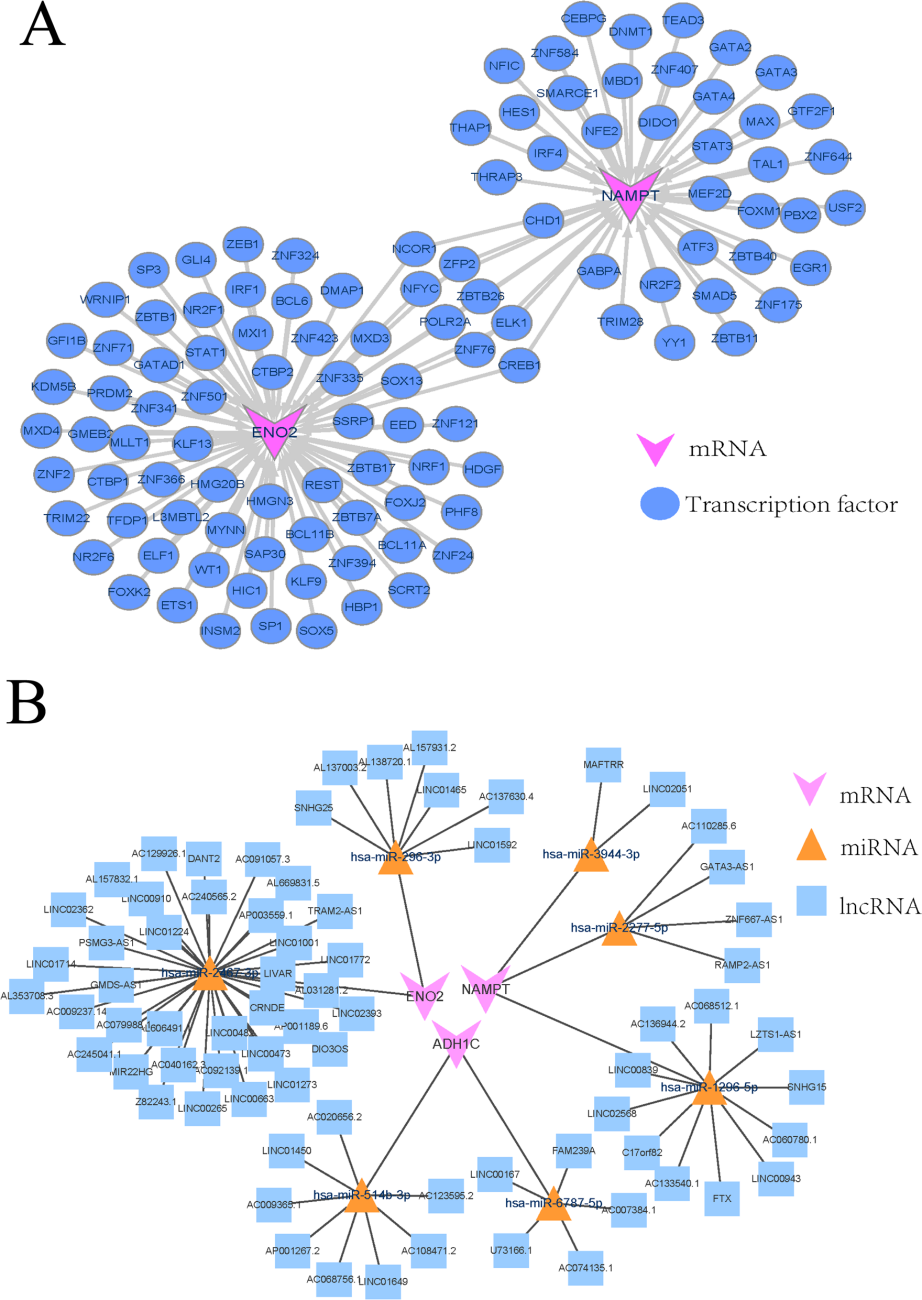
Biomarkers of the regulation network analysis diagram. **A** Diagnostic biomarkers of gene—TF control network. Pink inverted triangular cones represent genes, blue circles represent transcription factors, and arrows represent targeted regulatory relationships. **B** The lncRNA – miRNA—mRNA regulatory network of diagnostic biomarkers. Pink inverted triangular cones represent genes, yellow triangles represent miRNAs, and blue squares represent lncrnas

The Network Analyst database predicted the TF of biomarkers. 118 genes-F relationship pairs were obtained, including ENO2 and NAMPT, while ADH1C did not predict transcription factors (Fig. 4A). The prediction results of the targeting relationship show that there are 925 lncRNA-miRNA-RNA targeting relationship pairs. In addition, the miRNA network with the binding energy of miRNA and mRNA top2 was selected for visualization (Fig. 4B). The results show that the miRNA network related to ENO2 was composed of has-miR-467-p and has miR-96-p; The network composed of has miR-14b-p and has mir-6787-p were related to ADH1C, and the network composed of has miR-277-p, has miR-944-p, and has miR-944-p were related to NAMPT.

### Validation of expression and diagnostic efficiency of diagnostic biomarkers for glaucoma

**Fig. 5:**
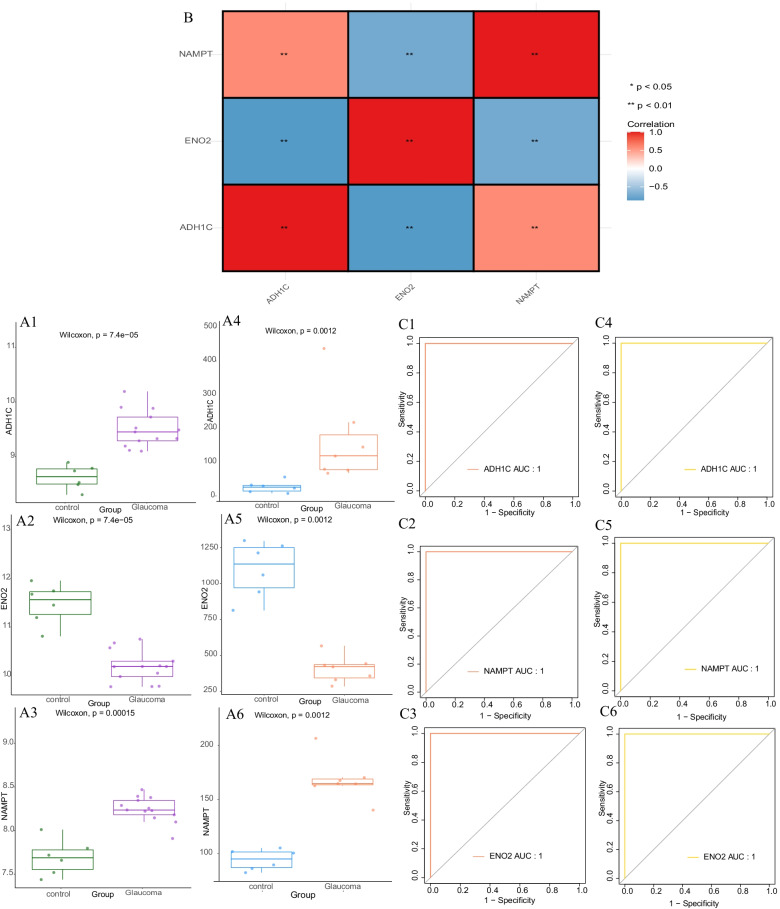
Diagnostic biomarkers expression and diagnostic value validation analysis. **A** Scatter plot of diagnostic biomarkers expression. GSE9944 data set (left), green said the normal control group, purple for glaucoma group. GSE2378 data set (right), Blue is the normal control group, orange said glaucoma group. **B** Heat map of correlation between glaucoma related marker genes. Red: positive correlation. Blue: negative correlation The darker the color, the greater the correlation. *: *P* < 0.05, * *: *P* < 0.01. **C** ROC curve validation of diagnostic biomarkers (C1 – C3: GSE9944 data set, C4 – C6: GSE2378 data set)

The expression of 3 biomarkers in the GSE9944 dataset suggested that NAMPT and ADH1C were up-regulated and ENO2 down-regulated in glaucoma samples. The validation results in the GSE2378 dataset were consistent with those in GSE9944 (Fig. 5A). Correlation analysis between biomarkers showed that ENO2 was negatively correlated with ADH1C (cor = -0.865714202) and NAMPT (cor = -0.730541227) (Fig. 5B). The ROC curve of the GSE 2378 dataset showed that the AUC of the three diagnostic biomarkers was 1 (Fig. 5C). The above results show that ENO2, NAMPT, and ADH1C have strong diagnostic values for glaucoma.

### Molecular docking between glaucoma diagnostic markers and compound with therapeutic activity for glaucoma

**Fig. 6:**
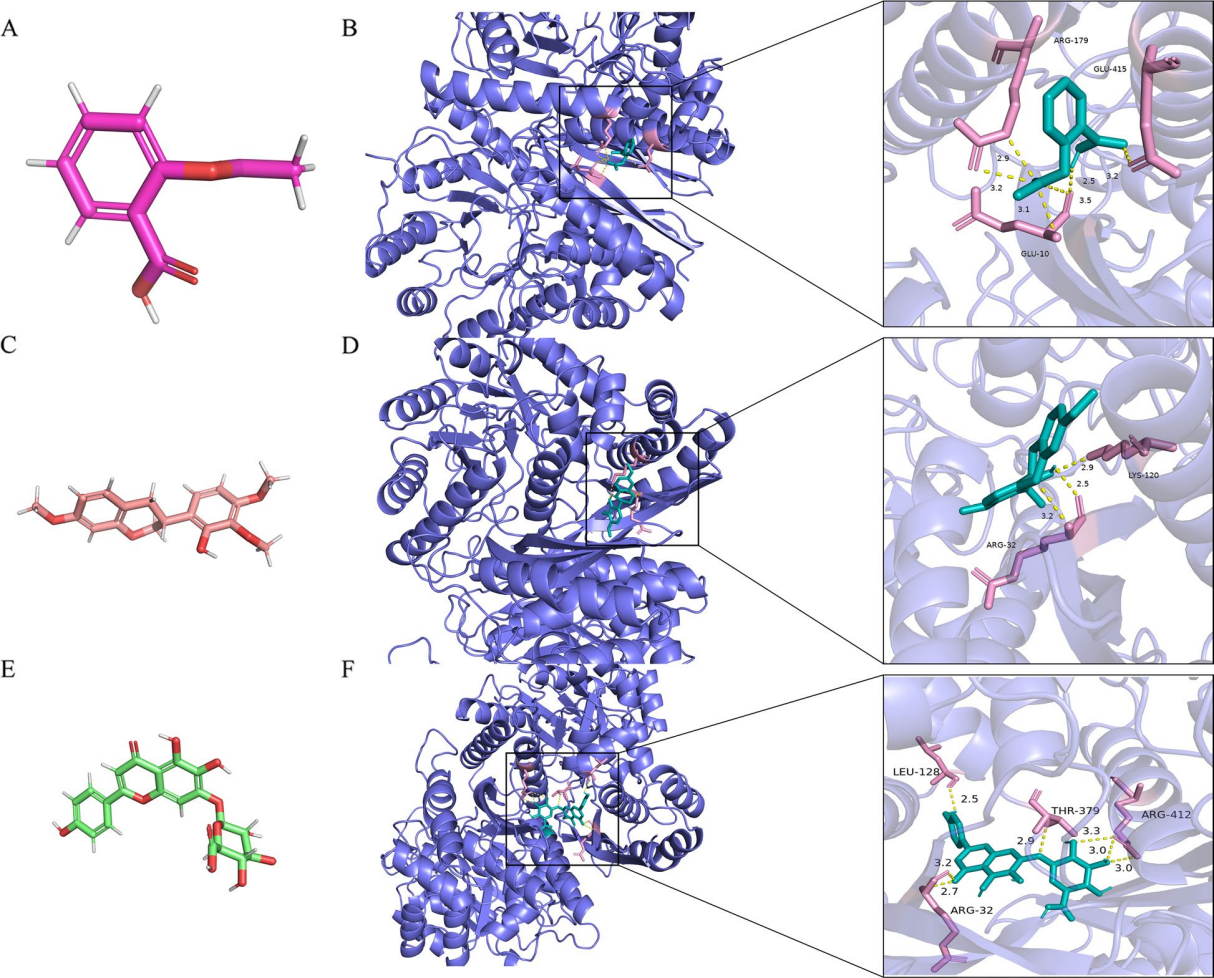
Olecular docking diagram between Diagnostic biomarker ENO2 and Acetylsalicylic acid, 7-O-Methylisomucronulatol and Scutellarin. **A** Schematic diagram of 3D conformer structure of acetylsalicylic acid. **B** Docking results of 4zcw and acetylsalicylic acid. **C** 3D Conformer structure of 7-O-Methylisomucronulatol. **D** Docking results of 4zcw and 7-O-Methylisomucronulatol. **E** 3D Conformer structure of Scutellarin. **F** Docking results of 4zcw and Scutellarin

The prediction results of GeneCards indicated that only ENO2 was included in the list of disease targets for glaucoma. In addition, 3 active molecules for glaucoma treatment were found in the TCMSP database (https://tcmspw.com/tcmsp.php): Acetylsalicylic acid [PMID: 34208432], 7-O-Methylisomucronulatol, Scutellarin [PMID: 34414202], and molecular docking was carried out with the diagnostic biomarker ENO2 (PDB ID of ENO2 is 4ZCW crystal structure). The molecular docking of Acetylsalicylic acid (3D converter structure, Fig. 6A) and ENO2 were carried out by Auto Dock vina (Fig. 6B). ARG-179, GLU-415, and GLU-10 residues have hydrogen bond interaction with the acetylsalicylic acid molecules. The docking affinity between active molecules and proteins was 5.5 kcal/mol (Fig. 6A, B). When molecular docking of ENO2 and 7-O-Methylisomucronulatol, in which LYS-120 and ARG-32 residues interact with 7-O-Methylisomucronulatol molecules by a hydrogen bond. The docking affinity between the active molecule and the protein was 7.3 kcal/mol (Fig. 6C, D). When molecular docking of ENO2 and Scutellarin among which ARG-32, ARG-412, LEU-128, and THR-379 residues interact with Scutellarin molecules by a hydrogen bond. The docking affinity between the active molecule and the protein was 8.9 kcal/mol (Fig. 6E, F).

## Discussion

The asymptomatic of glaucoma has a slow development process. Previous research indicated that the vision may have been seriously damaged before the early stage of glaucoma [13]. At present, many methods are used to diagnose, detect and screen, and point out the direction for clinical treatment of glaucoma. However, this has a great limitations on clinically available tools for analysis glaucoma. No gold standard, and it takes a long time of treatment and observation to confirm the clinical effect of treatment. Using biomarkers was conducive to the early diagnosis to better treatment and management of glaucoma. At the same time, the evaluation of glaucoma biomarkers also provides a basis for the discovery and research of glaucoma-targeted therapeutic drugs [14, 15]. Recently, artificial intelligence and spatial probability algorithms have high accuracy and data efficiency [16, 17]. Machine learning algorithm has been proved to be used for disease prediction, and among the Random Forest (RF) algorithm shows higher accuracy than the Support Vector Machine (SVM) algorithm and the Naïve Bayes algorithm, which provides advantages for the accurate diagnosis of glaucoma [18]. Glaucoma is only a generic term of a highly different disease group of optic neuropathies, which is characterized by high or normal intraocular pressure combined with damage to optic papilla, retinal nerve fiber layer, and glaucomatous visual field [19]. At the time of diagnosis, we comprehensively judge whether have glaucoma by intraocular pressure examination, atrial angle examination, optic disc structure examination, optic nerve evaluation, visual field, and visual function examination. In this study, the database of clinical diagnosis of glaucoma and normal samples in the database were used directly to study and diagnosed glaucoma from genetic aspects. Although the difference in age, gender, and race was taken into account, the influence of other factors on glaucoma was not considered due to data limitations. In our study, the glaucoma diagnosis model was constructed by machine learning algorithm LR-RF and LASSO algorithm, and 3 diagnostic biomarkers ADH1C, ENO2, and NAMPT were confirmed.

NAMPT and ADH1C were up-regulated in glaucoma samples, contrary ENO2 was down-regulated in glaucoma samples. NAMPT was closely related to nerve movement and survival. The loss of neuronal NAMPT will lead to motor neuron degeneration and functional defects in mice [20], and NAMPT has an important impact on human neurological diseases, this is consistent with the high expression of NAMPT in glaucoma samples [21, 22]. The development of glaucoma is associated with oxidative stress, ADH1C may be related to oxidative stress and mitochondrial dysfunction in glaucoma. The discovery of ADH1C may play a foundation for studying the pathogenesis of glaucoma from the aspects of oxidative emergency and antioxidation. The enzyme encoded by ENO2 exists in mature neurons and cells of neuronal origin, which is called neuron-specific enolase (NSE). At present, NSE was helpful to predict the visual acuity of primary open-angle glaucoma (POAG) patients [23, 24]. In addition, glaucoma target prediction indicated that only ENO2 existed in the database. ENO2 only exists in neurons, neuroendocrine and muscle tissues. ENO2 is closely related to neurodegenerative diseases [25]. The up-regulated expression of ENO2 in NSCLC indicated that the ENO2 can be used as a marker [26, 27]. The serum of NAMPT level of patients with a history of retinal vascular occlusion (RVOs) was much lower than that of healthy individual. NAMPT-PBEF-visfatin serum levels can be used as a marker of RVOs [28].

The regulatory network of transcription factor and non-coding RNA regulatory of glaucoma in diagnostic markers reveal the expression regulation mechanism of these three diagnostic markers at the transcription and post-transcriptional levels, respectively. Studies have shown that transcription factors can be used as the diagnostic marker or the key to optic nerve degeneration in glaucoma. For example, SIX6 risk variation, silent information regulator T (SIRT) 1, and Forkhead Box O (FOXO) transcription factor 1 and 3a ( FOXOs 1 –3a), which is the key to the pathogenesis of glaucoma or optic nerve degeneration [29, 30]. Furthermore, the target gene and its relationship with miRNA and signal pathway can realize the diagnosis of glaucoma [31], lncRNA and miRNA have also been proved to be key to glaucoma diagnosis and retinal nerve regulation. For example, lncRNAs (DNAJC27-AS1, AF121898, OIP5-AS1, and SNX29P2) were established as hub RNAs in the ceRNA network of POAG [32]. The expression levels of lncRNAs T267384, ENST00000607393, and T342877 as biomarkers of glaucoma [33]. The 20 miRNAs as potential biomarkers of glaucoma have been found [34], the isolation and inhibition of mir-615 were considered to have a certain effect on retinal neurodegeneration [35]. Our research shows that ENO2 and NAMPT are regulated by a large number of transcription factors, among them, there are 8 common transcription factors. CeRNA network analysis of the three diagnostic markers obtained a total of 925 lncRNA-miRNA-mRNA targeting pairs. The network sorted according to the binding energy of miRNA and mRNA, ENO2, has-miR-2467-3p and has-miR-296-3p, ADHIC, has miR-514b-3p and has miR-6787-5p, and NAMPT has miR-2277-5p, has miR-3944-5p, and has miR-3944-3p.

To explore whether the three diagnostic molecules can be used as therapeutic targets of glaucoma. Three compounds with glaucoma therapeutic effects were selected by the TCMSP database, which was used for molecular docking with diagnostic markers. 7-O-Methylisomucronulatol was key compound in the compound-target network of Qing Guang An Granule (QGAG) to the treatment of glaucoma [36]. Scutellarin was discovered as a substance that protects the nerves of the eyes and brain, which is a new neurotherapeutic agent for glaucoma treatment, it has been studied in mice, at present, [37, 38]. Acetylsalicylic acid has complex effects on the changes of intraocular pressure, studies have demonstrated that single-use of acetylsalicylic acid cannot significantly reduce intraocular pressure in glaucoma patients, but long-term use will have an impact on intraocular pressure [39, 40]. There was a distinguished effect that acetylsalicylic acid is used in combination with other drugs to treat glaucoma [41]. The glaucoma-related gene list in GeneCards only contains ENO2 among the three diagnostic markers, ENO2 was used for molecular docking. These results indicate that ENO2 may be a potential target for glaucoma.

## Conclusion

Our research shows that ENO2, NAMPT, and ADH1C as diagnostic markers have been proved to be effective in the diagnosis of glaucoma, and ENO2 is a potential therapeutic target.

## Supplementary Information

**Additional file 1.**

**Additional file 2.**

**Additional file 3.**

## Data Availability

The available data has been placed in the Supplementary materials. Web links and URLs: The GEO database (https://www.ncbi.nlm.nih.gov/geo/). The network analyst (https://www.networkanalyst.ca/) database. The mirwalk (http://mirwalk.umm.uniheidelberg.de/) database. The Starbase (http://starbase.sysu.edu.cn/). Gene cards (https://www.genecards.org/) database. The TCMSP database (https://tcmspw.com/tcmsp.php). The KEGG database (https://www.kegg.jp).

## Abbreviations

LR: Logistic Regression
RF: Random Forest
LASSO: Lasso regression
TF: Transcription factor
SVM: Support Vector Machine
NSE: Neuron specific enolase
POAG: Primary open angle glaucoma
RVOs: Vascular occlusion
SIRT: Silent information regulator
FOXO: Forkhead Box O
QGAG: Qing Guang An Granule
