# Supplementary material for: Based on multiple machine learning to identify the ENO2 as diagnosis biomarkers of glaucoma
Source: BMC Ophthalmol. 2022 Apr 2;22:155. doi: 10.1186/s12886-022-02350-w (PMC8976990; doi:10.1186/s12886-022-02350-w)
Supplement: Supplementary file 2 — Additional file 2. [file 12886_2022_2350_MOESM2_ESM.pdf]

	Total (N=13)	GSE2378 glaucoma (N=7)	GSE2378 normal (N=6)	P-value
**eye**				
A eye	1 (7.7%)	1 (14.3%)	0 (0%)	0.629
left eye	6 (46.2%)	3 (42.9%)	3 (50.0%)	
right eye	6 (46.2%)	3 (42.9%)	3 (50.0%)	
**gender**				
female	7 (53.8%)	4 (57.1%)	3 (50.0%)	1
male	6 (46.2%)	3 (42.9%)	3 (50.0%)	
**age (years)**				
>=60	10 (76.9%)	6 (85.7%)	4 (66.7%)	0.879
<60	3 (23.1%)	1 (14.3%)	2 (33.3%)	
