# Supplementary figures and images for: Based on multiple machine learning to identify the ENO2 as diagnosis biomarkers of glaucoma
Source: BMC Ophthalmol. 2022 Apr 2;22:155. doi: 10.1186/s12886-022-02350-w (PMC8976990; doi:10.1186/s12886-022-02350-w)

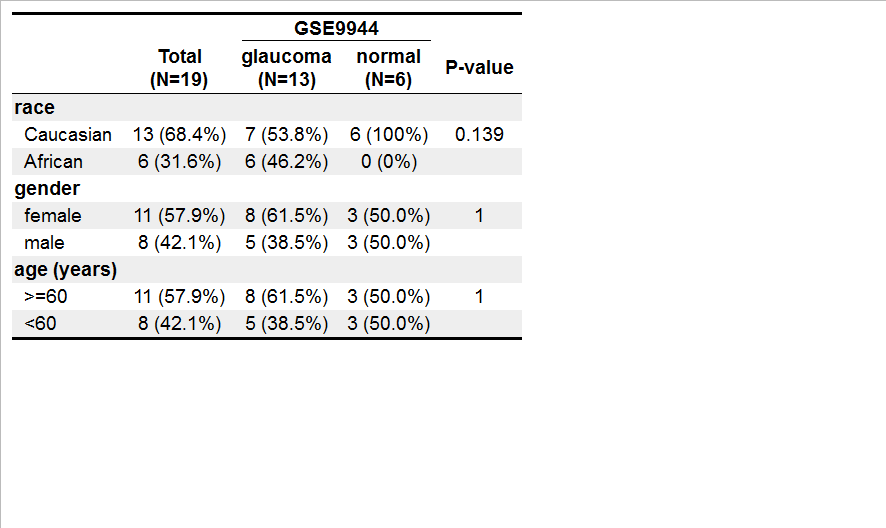

Supplement: Supplementary file 3 — Additional file 3. [file 12886_2022_2350_MOESM3_ESM.png]
